# Ligand field molecular dynamics simulation of Pt(II)-phenanthroline binding to N-terminal fragment of amyloid-β peptide

**DOI:** 10.1371/journal.pone.0193668

**Published:** 2018-03-06

**Authors:** Matthew Turner, Shaun T. Mutter, Robert J. Deeth, James A. Platts

**Affiliations:** 1 School of Chemistry, Cardiff University, Park Place, Cardiff, United Kingdom; 2 Department of Chemistry, University of Warwick, Gibbet Hill, Coventry, United Kingdom; Torrey Pines Institute for Molecular Studies, UNITED STATES

## Abstract

We report microsecond timescale molecular dynamics simulation of the complex formed between Pt(II)-phenanthroline and the 16 N-terminal residues of the Aβ peptide that is implicated in the onset of Alzheimer’s disease, along with equivalent simulations of the metal-free peptide. Simulations from a variety of starting points reach equilibrium within 100 ns, as judged by root mean square deviation and radius of gyration. Platinum-bound peptides deviate rather more from starting points, and adopt structures with larger radius of gyration, than their metal-free counterparts. Residues bound directly to Pt show smaller fluctuation, but others actually move more in the Pt-bound peptide. Hydrogen bonding within the peptide is disrupted by binding of Pt, whereas the presence of salt-bridges are enhanced.

## Introduction

Alzheimer’s disease (AD) is one of the greatest healthcare challenges facing modern society.[1] Its aetiology is complex, but the importance of amyloid-β (Aβ) peptides and their aggregation is well established.[2][3][4][5] AD is associated with formation of fibrils and plaques (dense, insoluble deposits of protein and cellular material outside and around neurons) in brain tissue that impair proper functioning of neurons. Plaques are formed by aggregation of Aβ that are soluble in isolation, but insoluble when bound to one another. The presence of metals, notably copper, zinc and iron, is a vital part of the aggregation and subsequent toxicity of Aβ: increased levels of Cu and Zn are found in plaque regions of diseased brain, [6][7] and those plaques which do not contain metal ions have been found to be non-toxic.[8] Moreover, platinum complexes inhibit aggregation,[9][10][11] opening new avenues for treatment and diagnosis. In these, ligand choice proves to be vital, with large planar aromatic groups acting to stabilise complexes between Pt and Aβ.[9][12][13]

Structural details for naturally occurring metals such as Cu and Zn have been elucidated through a wide range of experimental [14][15] and simulation techniques,[16][17][18][19] but the equivalent for Pt is scarcer. Ma et al used HPLC, ESI-MS and NMR spectroscopy to examine the binding of Pt^II^-phenanthroline to Aβ, suggesting that binding to His6 and His14 predominates and that π-stacking to aromatic residues Phe4, Tyr10 and His13 may also play a role.[20][21] In addition, Streltsov et al used a combination of EXAFS and DFT to probe local binding environment at Pt and derive structural models for the interaction of platinum complexes with Aβ16 and Aβ42.[22] Recently, we showed that ligand field molecular mechanics (LFMM) [23][24][25] is an appropriate method to probe the binding of Pt to fragments of Aβ, characterising the effect of Pt complexes on limiting conformational freedom of the peptide [26] and the role of ligand variation in complexes formed and 3D conformation adopted. [27] In this work, we extend this approach to examine the dynamical behaviour of a typical Pt-Aβ adduct using molecular dynamics simulations and LFMM description of metal coordination coupled with conventional molecular mechanics (MM) for the peptide.

## Computational details

Molecular Dynamics simulations were carried out using a modified version of DL_POLY 2.0 [28] that incorporates LFMM energies and forces.[29] Pt(Aβ) complexes were described using a combination of LFMM for Pt(II) [29][30] and AMBER94 [31] parameters for all other atoms. The Aβ1–16 peptide was built in extended conformation in MOE,[32] and protonation states at pH 7.4 assigned using the Protonate3D module of this package. Pt-phenanthroline complexes were bound to the peptide via His6-Nε and His14-Nε, as identified in our previous work.[26][27] Initial peptide conformations were selected from a LowMode Molecular Dynamics [33] simulation in MOE as reported previously. DL_POLY input files were generated using DL_FIELD [34] and the DommiMOE [24] extension to MOE.

For all simulations of the free Aβ peptide, AMBER94 partial charges assigned by MOE were used. For Pt(phen)-Aβ simulations, Merz-Kollman charges were calculated for model Pt(phen)-imidazole systems from HF/6-31G(d)/SDD electrostatic potential in Gaussian09,[35] with Pt^II^ given a van der Waals radius of 2.0 Å (see ESI). The remaining peptide atoms were assigned AMBER94 charges as calculated by MOE.

Simulations were performed on isolated systems, with reaction field solvation in dielectric constant 78.4. Simulations were performed in the NVT ensemble, where temperature was controlled at 310 K using the Nosé-Hoover thermostat [36][37] with relaxation constant 0.5 ps. Equations of motions were integrated using a Verlet Leapfrog algorithm, with a timestep of 1 fs. The SHAKE algorithm [38] with tolerance 10^−8^ Å was used to constrain bonds containing hydrogen. The vdW forces were calculated with a cutoff of 1 nm, while 2.1 nm was used as a cutoff for electrostatics. In each molecular dynamics trajectory, atomic positions and velocities were recorded every 500 fs and used for subsequent analysis.

Resulting MD trajectories were analysed using VMD, [39] with root mean square deviation (RMSD), radius of gyration, peptide secondary structure, hydrogen bonds, salt bridges, solvent accessible surface area and RMSF data recorded. The STRIDE algorithm [40], as implemented in VMD, was used to characterise each residue as either Turn, β-sheet, β-bridge, α-helix, 3_10_-helix, π-helix or Coil- type structure.

## Results and discussion

**Fig 1:**
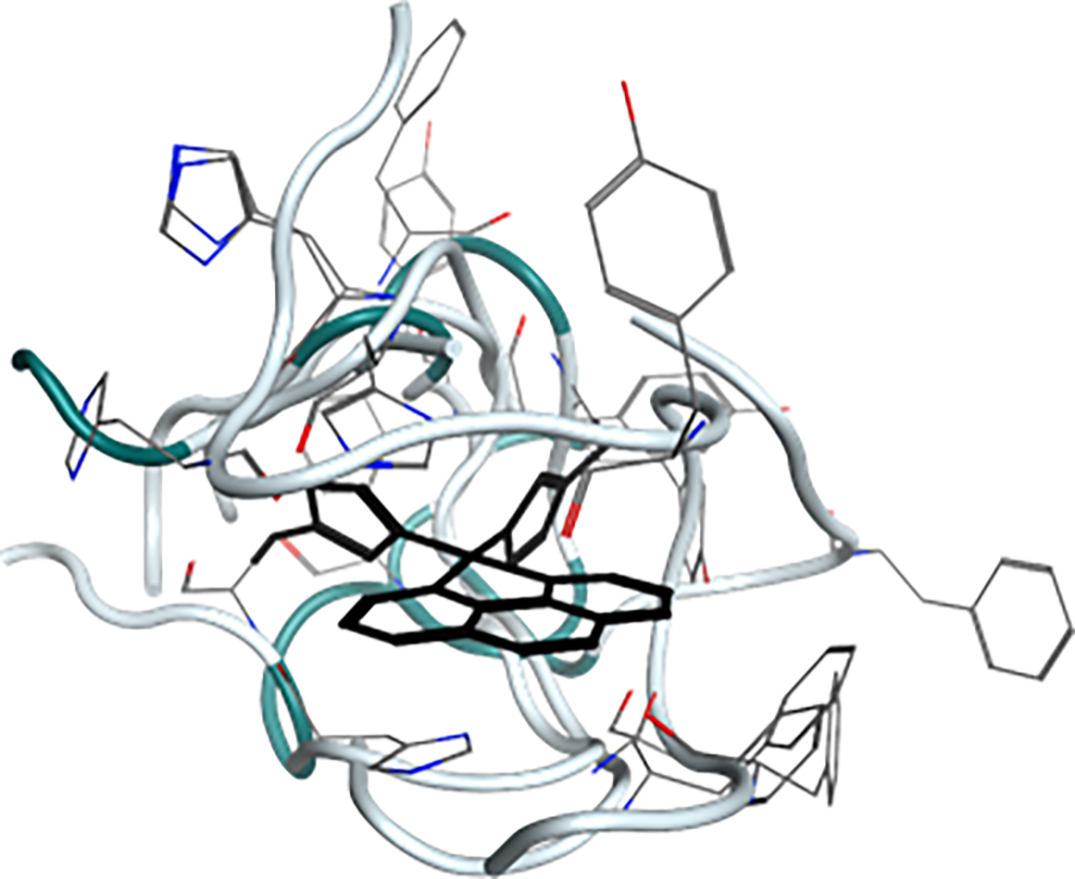
**Starting points of simulations F–J.** Pt(phen) aligned, backbone shown as tube, sidechains as wireframe and Pt(phen)(imid)_2_ in black.

**Fig 2:**
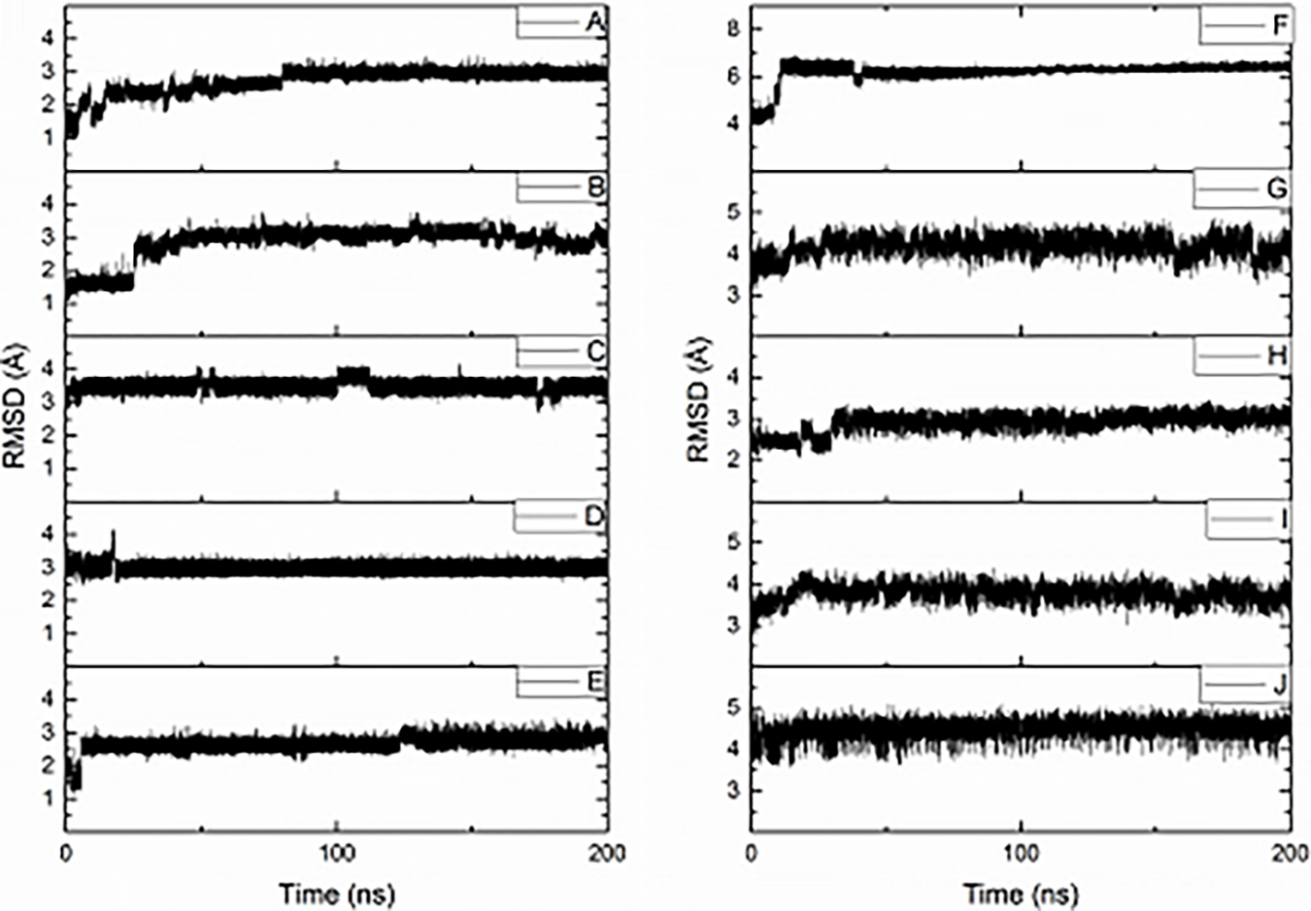
**RMSD vs time for simulations A-J**.

Five simulations (labelled A-E) of the free Aβ16 fragment were carried out, each for 200 ns, along with five simulations (labelled F-J) for the Pt(Aβ16) system. Table 1 illustrates that all initial conformations are significantly different from one another, ensuring efficient sampling of the molecular phase space during simulations. Fig 1 shows an overlap of starting points of simulations F–J, showing the variation of backbone and sidechain conformations adopted. Equilibration of all ten simulations was monitored initially via the RMSD relative to starting points, as shown in Fig 2. Following recent work on Aβ40 [41] and Aβ42 [18], we consider that these simulations are ‘pseudo-equilibrated’ as RMSD fluctuates around a central point by approximately 1 Å after a period of time taken as 80, 40, 10, 20 and 10 ns for A-E, 50, 25, 30, 25 and 25 ns for F-J, respectively. Simulation data beyond these points were used for further analysis.

**Table 1:** RMSD between starting points of free and platinated peptides.

RMSD (Å)	A	B	C	D	E		F	G	H	I	J
A	-	2.140	4.420	6.459	8.213	F	-	7.605	6.334	7.485	6.323
B	-	-	4.310	6.362	8.259	G	-	-	6.400	3.301	5.949
C	-	-	-	5.488	7.261	H	-	-	-	5.774	5.807
D	-	-	-	-	5.763	I	-	-	-	-	5.797
E	-	-	-	-	-	J	-	-	-	-	-

Table 2 summarises RMSD relative to the initial configuration for all ten sets of production MD runs. Free peptide values vary between 2.7 and 3.5 Å, with standard deviations in the range 0.1 to 0.2 Å, indicating relatively small movement from starting point and hence at least pseudo-equilibration of the individual simulations. Platinated peptides exhibit larger changes, equilibrating to between 3.0 and 6.3 Å, with maximum values also rather larger than those seen for the free peptide but standard deviations of similar magnitude, suggesting that these structures fluctuate to a similar degree. Moreover, RMSD values vary rather more between different simulations than within each one (A-E: mean = 3.03 Å, sd = 0.30 Å; F-J: mean = 4.36 Å, sd = 1.22 Å). This highlights the importance of using multiple starting points, since any one simulation reaches pseudo-equilibration relatively quickly and does not visit the entire configurational phase space available to the peptide.

**Table 2:** Mean, standard deviation, maximum and minimum RMSD relative to starting point for production MD data (Å).

	Mean	SD	Min.	Max.
A	2.946	0.084	2.535	3.514
B	3.043	0.162	2.299	3.774
C	3.503	0.140	2.671	4.182
D	2.969	0.088	2.549	3.485
E	2.686	0.149	2.090	3.449
F	6.292	0.101	5.68	6.777
G	4.224	0.192	3.251	4.887
H	2.983	0.135	2.413	3.471
I	3.794	0.166	3.013	4.382
J	4.501	0.176	3.628	5.092

**Fig 3:**
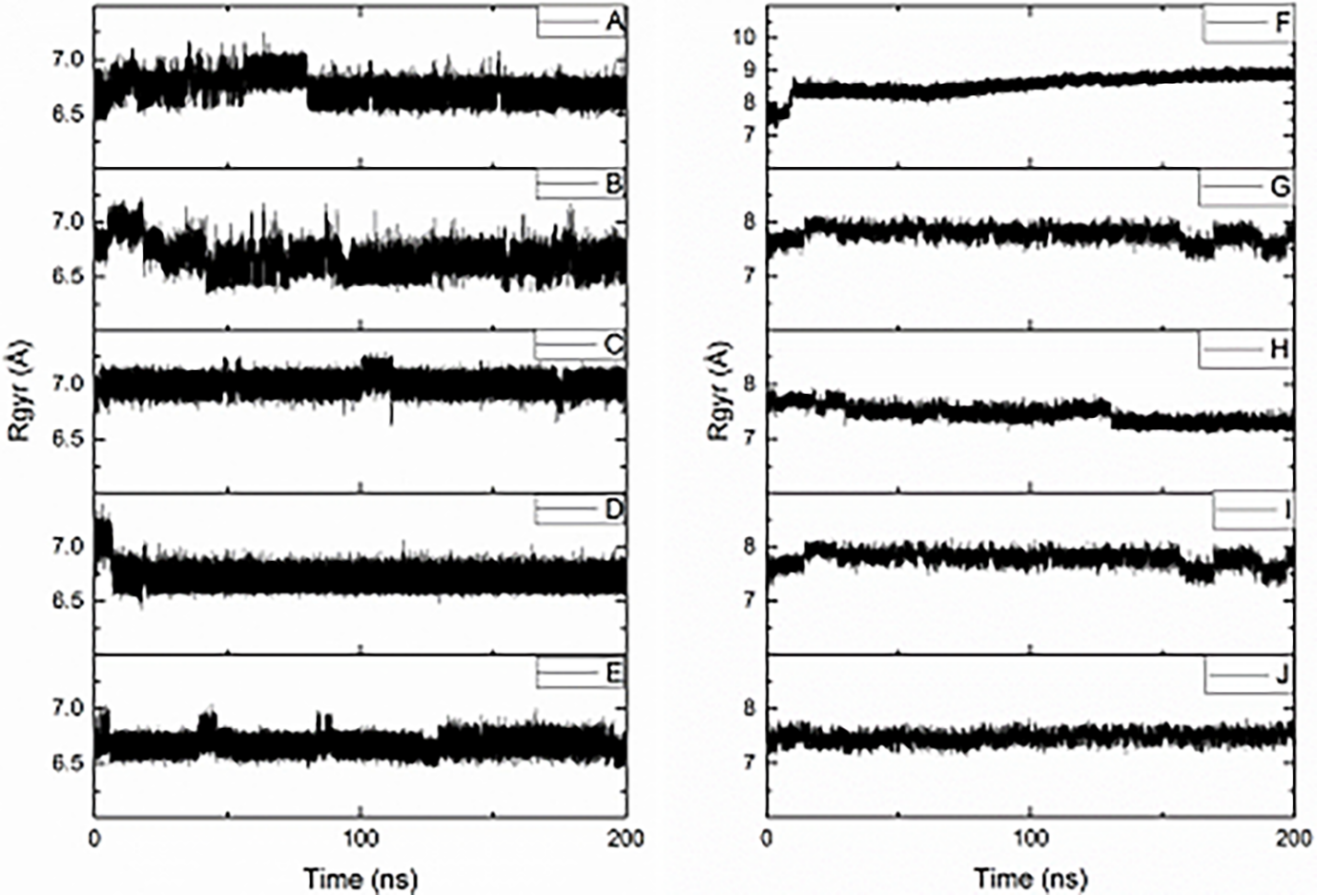
**Radius of gyration of simulations A–J**.

Fig 3 shows radius of gyration (R_g_) data for ten simulations, with post-equilibration data summarised in Table 3. These show that the free Aβ16 fragment adopts a relatively compact structure, with R_g_ values typically less than 7 Å: for comparison, Aβ16 in an extended conformation has R_g_ of 16.99 Å, and in α-helical structure, 9.16 Å. Possible intra-molecular interactions that might give rise to these compact conformations are considered below. The standard deviation within each simulation is also small, indicating that there is little change in the compactness of the peptide structure during any one simulation. In general, different simulations display a similar range of R_g_ values (A-E: mean = 6.74 Å, sd = 0.15 Å; F-J: mean = 7.83 Å, sd = 0.50 Å). This suggests that while the peptide as a whole remains compact, flexible peptide side-chains may be responsible for the similar degree of variation observed. However, simulation C has slightly larger average R_g_ value than the others: interestingly, C also displayed the greatest average RMSD value of the Aβ16 systems studied, suggesting that this simulation occupies a somewhat different configuration than the others.

**Table 3:** Mean, standard deviation, maximum and minimum radius of gyration for production MD data (Å).

	Mean	SD	Min	Max
A	6.695	0.060	6.456	7.084
B	6.627	0.095	6.324	7.178
C	6.998	0.057	6.610	7.291
D	6.706	0.051	6.483	7.060
E	6.675	0.058	6.450	7.043
F	8.659	0.196	7.932	9.173
G	7.786	0.125	7.252	8.165
H	7.417	0.126	7.085	7.812
I	7.786	0.125	7.252	8.156
J	7.481	0.095	7.121	7.831

Pt(Aβ16) simulations display significantly larger mean R_g_ values than their free peptide counterparts, indicating that coordination of the large, sterically demanding Pt-phen forces the structure to adopt less compact conformations. Standard deviation is consistent across these simulations, though slightly larger than for the free peptide systems. Pt(Aβ16) simulations are centred around 3 distinct R_g_ values, *ca*. 7.45 Å, 7.78 Å and 8.66 Å. F displays the largest R_g_ of all Pt(Aβ16) simulations (8.66 ± 0.2 Å), significantly larger than all other conformations examined. Interestingly, this conformation displays a high prevalence of stabilising π-π interactions (*vide infra*), which may cause the peptide backbone to extend and re-organise in order to accommodate these stacking arrangements. Simulations G and I display identical R_g_: comparison of the final snapshot of the each trajectory shows that the structures are near-identical (RMSD = 0.11 Å compared with 3.30 Å at starting points), showing that the simulations converged to a common structure within the equilibration period.

**Fig 4:**
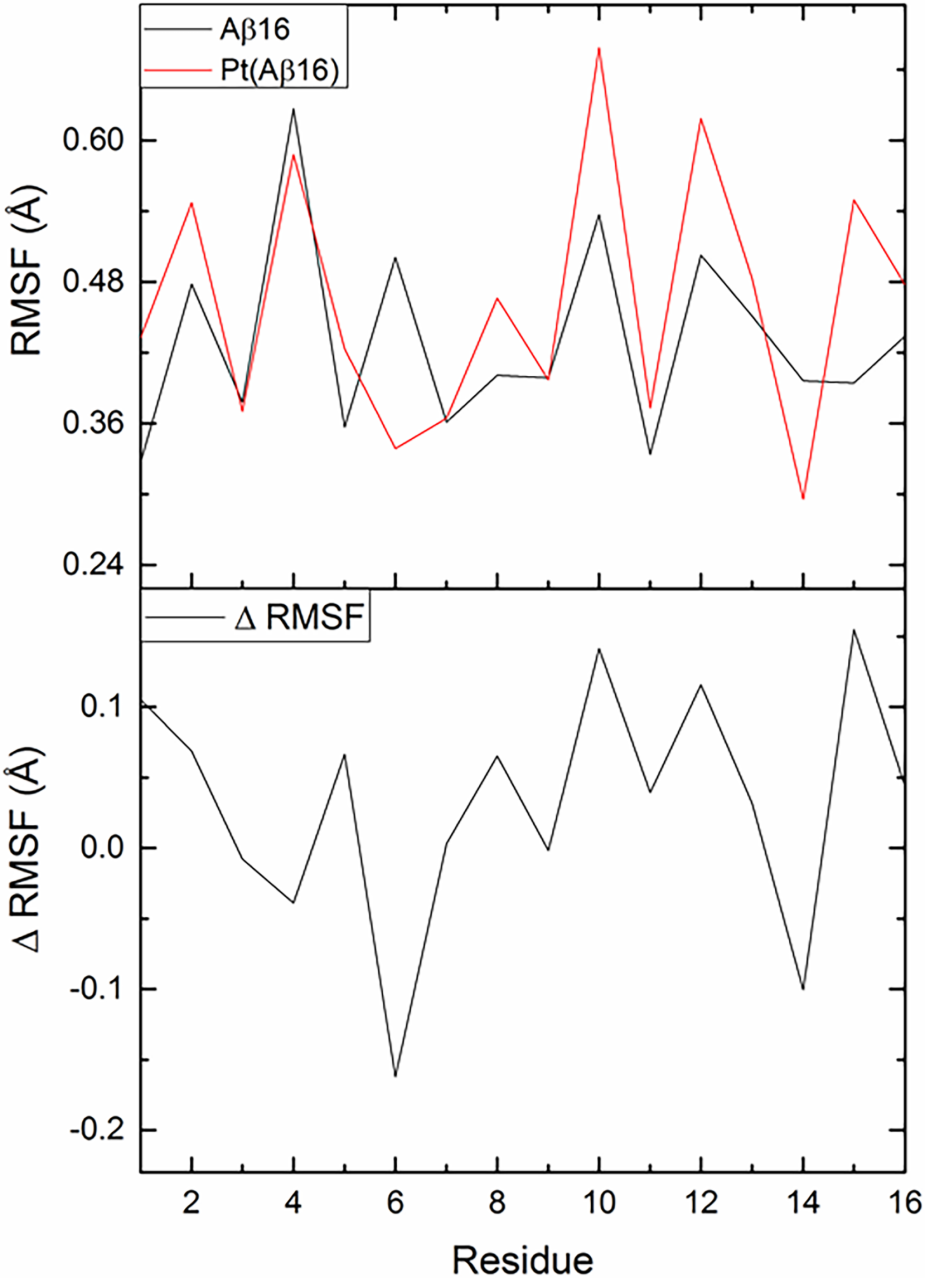
Average RMSF for Aβ16 and Pt(Aβ16), and the difference between average values.

Root mean square fluctation (RMSF) of each residue was measured for production MD, as shown in Fig 4 (data from individual trajectories are shown in ESI). For the free peptide, large RMSF values are observed for Phe4, His6, Tyr10 and Val12, and small values for Asp1, Glu3, Arg5, and Glu11. Coordination of Pt(phen) at histidines 6 and 14 unsurprisingly reduces their RMSF, with Phe4 also moving less than in the metal-free case, whereas values for Tyr10, Val12 and Lys15 are on average larger after metallation. These data therefore suggest that coordination of Pt(phen) affects the peptide in more subtle ways than might first be thought, in particular promoting more flexibility in resiudes lying between coordination sites. However, differences between free and Pt(phen) trajectories are of similar magnitude to those between repeat simulations. RMSF for Tyr10, for instance, is relatively constant at 0.53 ± 0.09 Å over 5 simulations of the free peptide, but varies rather more (0.67 ± 0.31 Å, max = 0.95 Å, min = 0.29 Å) for the equivalent Pt(phen) trajectories. Only three residues (His6, His14 and Gln15) exhibit differences in RMSF that exceed the sum of standard deviations: full details can be found in Supporting Information.

**Fig 5:**
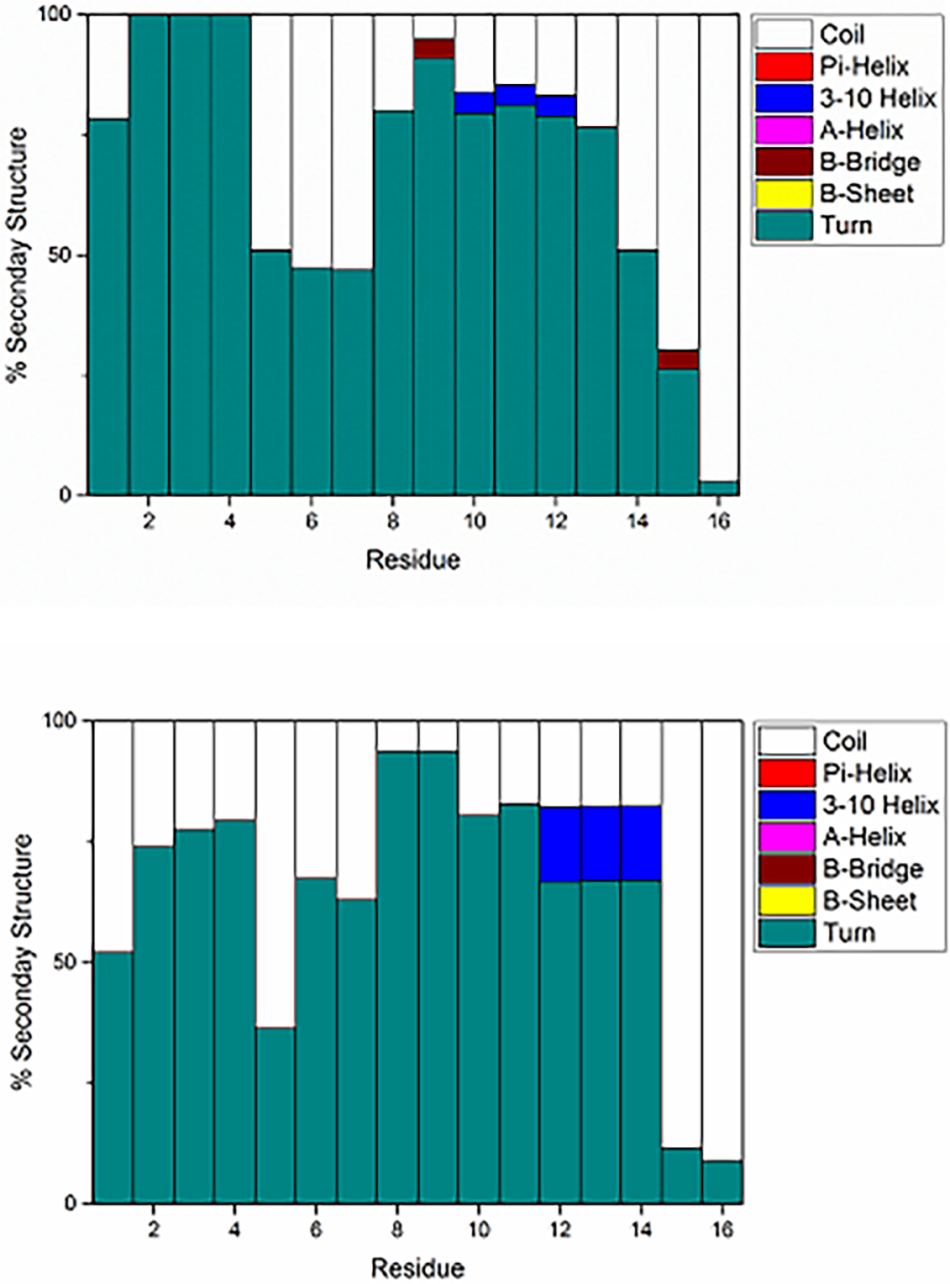
**Percentage secondary structure of Aβ16 (top) and Pt(Aβ16) (bottom)**.

In addition, the effect of Pt^II^(phenanthroline) coordination on secondary structure was determined. Percentages of each type of structure over all simulations is shown in Fig 5, with numerical values reported in Supporting Information. As expected for an intrinsically disordered peptide, most residues in the free peptide adopt coil or turn conformations. Interestingly, there is a low propensity (4% of total simulation time) for residues Tyr10-Glu11-Val12 to form a 3 residue 3,10 helix, while residues Gly9 and Gln15 infrequently (4% of simulation) adopt β-bridge structures. The prevalence of turn and coil structures in these simulations is in good general agreement with most data for Aβ, though the propensity of residues to adopt turn structures is high. Other authors have noted that STRIDE has a greater tendency to assign turn structure than other secondary structure programs [42], but observed that most residues in Aβ exist as turn structure with approximately 30–80\% probability. In addition, previous simulations of Aβ42 [42] displayed 3,10-helical structure for residues Tyr10-Val12 of approximately 3–5%, in agreement with our data.

In the Pt(Aβ16) simulations, secondary structure is again predominantly assigned as either turn or coil, but the percentages of each secondary structure element differs from the free peptide. This indicates that coordination of Pt(phen) does not drastically change secondary structure, although Pt coordination shifts the 3,10-helix towards the C-terminus and increases its incidence to 15% of total simulation time. Strikingly, residues involved in metal binding (His6, His14) show an increase in defined secondary structure (turn and helix) in the Pt(Aβ16) simulations, from approximately 50% in Aβ16 to 60–80% in the platinated system.

Hydrogen bond networks within free and platinated Aβ16 were also monitored (Table 4). In both cases there is a large variation across trajectories, with as few as zero and as many as 17 (free) or 13 (platinated) present in production data. In general, the Pt(Aβ16) systems show fewer hydrogen bonds than the free Aβ16 simulations, suggesting that the Pt(phenanthroline) system may interfere with the natural hydrogen bonding patterns of Aβ16. However, this difference is not significant, given the large standard deviations (~1.9–2.0) on the mean number of hydrogen bonds.

**Table 4:** Mean, standard deviation, maximum and minimum number of hydrogen bonds in trajectories A–J.

	Mean	SD	Min	Max
(A)	6.43	1.99	0	16
(B)	5.61	1.86	0	14
(C)	7.05	1.86	0	17
(D)	7.11	2.06	0	17
(E)	5.78	1.81	0	15
(F)	5.70	1.66	0	13
(G)	5.15	1.74	0	13
(H)	3.86	1.64	0	12
(I)	5.15	1.75	0	12
(J)	5.95	1.78	0	13

**Fig 6:**
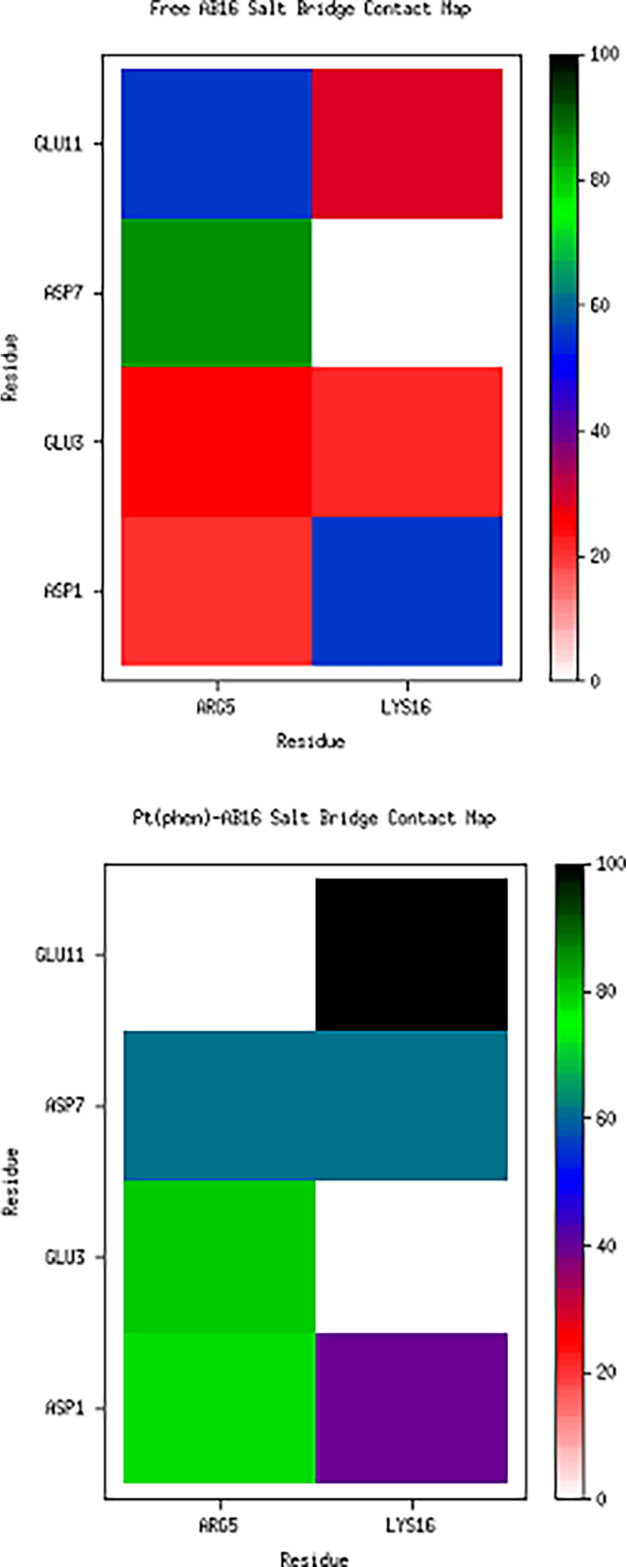
**Percentage of frames containing specified salt bridges, averaged over trajectories A–E (top) and F–J (bottom)**.

Intramolecular salt bridges, known to be important in the formation of fibrils,[43]^,^[44] were monitored during the course of the simulations. As the Aβ16 peptide fragment has two positively charged and four negatively charged residues, there are a total of eight possible salt bridge interactions. Each was monitored in VMD, defined as contact of less than 3.2 Å between O/N atoms in charged residues. Resulting data are summarised in Fig 6 and Table 5.

**Table 5:** Percentage of frames containing specified salt bridges, averaged over all trajectories A–E and F–J.

Aβ16	Asp1	Glu3	Asp7	Glu11
Arg5	20.99	25.93	85.64	55.88
Lys16	55.90	21.39	0.00	28.57
Pt(Aβ16)	Asp1	Glu3	Asp7	Glu11
Arg5	77.62	79.71	61.26	0.00
Lys16	39.57	0.00	61.91	99.44

In the free Aβ16 peptide, Arg5 predominantly interacts with Asp7 (86% of frames) but also forms interactions with Glu11 (56%) and less frequently with Glu3 and Asp1. Lys16 forms salt bridges most frequently with Asp1 (56%), and also interacts with Glu3 and Glu11 (20–30%). In all trajectories, Lys16 never forms a salt bridge with Asp7. Binding of Pt(phen) induces clear changes in salt bridge structure: Arg5 still interacts with Asp7 but with reduced frequency (61%), and instead primarily interacts with Glu3 and Asp1 (*ca*. 80%), while no Arg5-Glu11 interactions are present. Lys16 forms a near-constant salt bridge with Glu11 (99%), as well as frequent interactions with Asp7 (62%) and Asp1 (40%). In contrast to the free peptide, Lys16 does not form a salt bridge at all with Glu3 in the Pt(Aβ16) simulations. Overall, Pt coordination causes Arg5 to switch from bridges with Asp7 and Glu11 to Glu3 and Asp1, while Lys16 salt bridges are formed with Glu11 and Asp7 instead of Asp1. It is notable that the combined percentage of observed salt bridge interactions exceeds 100%, indicating that residues are close enough to their charged partners to simultaneously form multiple salt bridge interactions.

**Fig 7:**
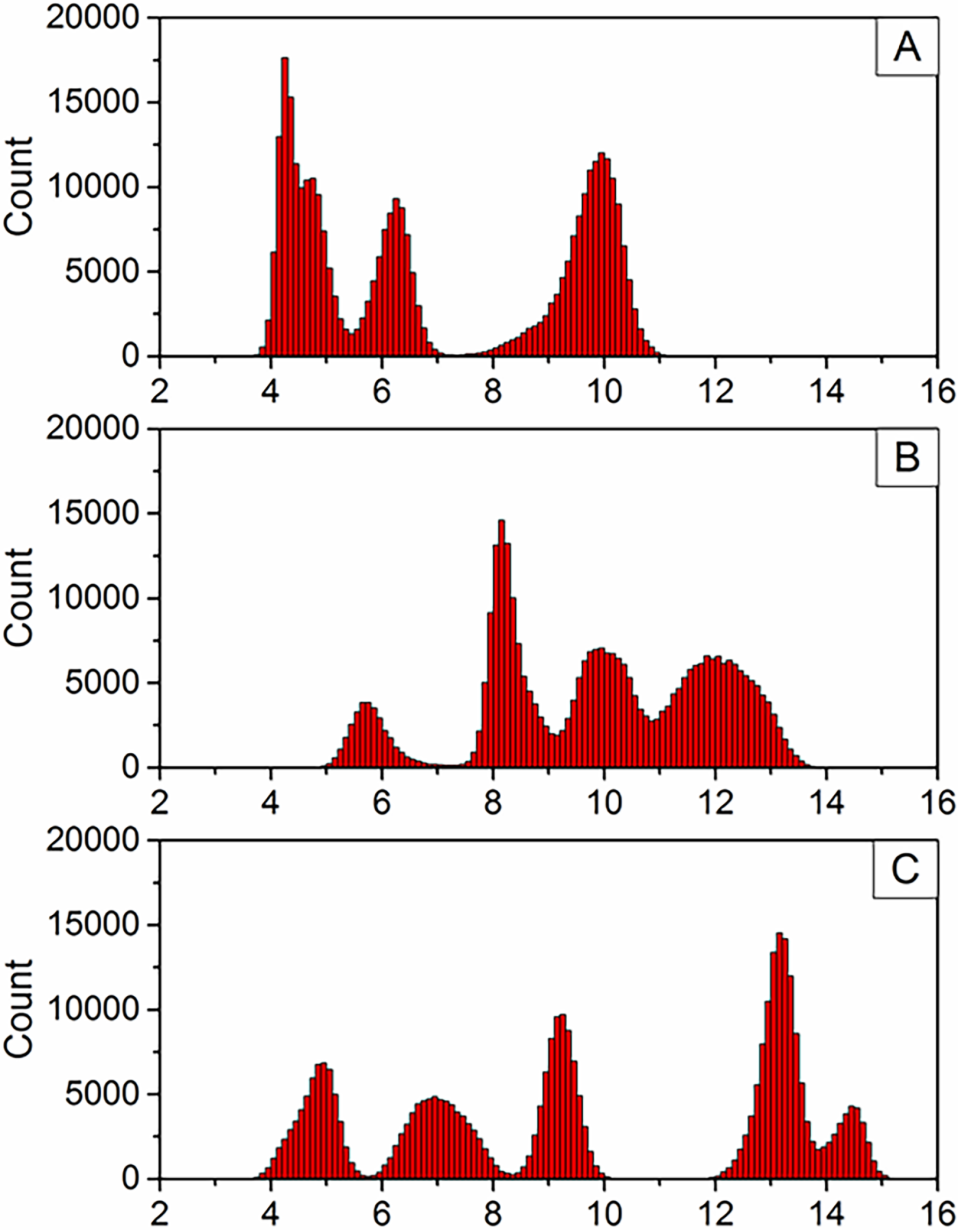
**Distribution of phen contacts with (A) Phe4, (B) Tyr10 and (C) His13 (Å)**.

**Fig 8:**
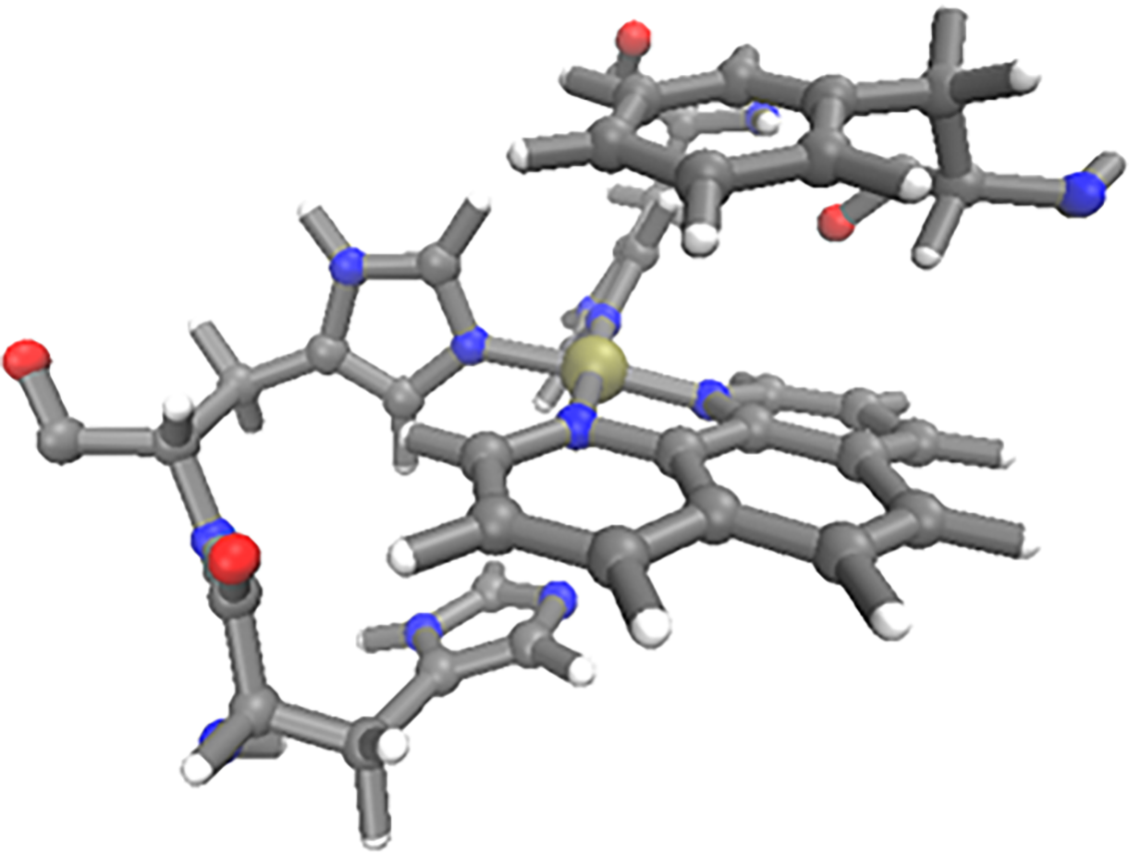
Detail from final frame of trajectory F, showing close contact of Phe4 and His13 with phenanthroline.

π-π stacking interactions between ligand and aromatic side chains are thought to be important in targeting coordination to the N-terminus of Aβ, as well as stabilising adducts. Close contacts between phen and side chains of Phe4, Tyr10 and His13 (defined as distance between Cγ and central C in phen) were monitored over simulations F–J. Distribution of these contacts are shown in Fig 7, showing that Phe4 in particular forms frequent π-π interactions with the ligand, with a high distribution of contacts < 5 Å. Tyr10 forms almost no π-π interactions with the phenanthroline ligand, with only a few frames at distances of approximately 5–6 Å, while His13 shows moderate distribution of states where the π-π inter-plane distance is less than 5 Å, sufficient for weak π-π interaction. Of particular interest is simulation F, where both Phe4 and His13 simultaneously form π-π stacking arrangements with the ligand for the final 150 ns of the simulation, with one residue above and below the ligand plane, as shown in Fig 8. These findings are in agreement with the findings of Ma et al [20][21], who reported stacking interactions with side chains of aromatic N-terminal residues, although the dynamical nature of our simulations reflect the highly flexible, intrinsically disordered nature of the Aβ peptide.

## Conclusions

Molecular dynamics, using LFMM description of metal coordination coupled with AMBER description of peptide, elaborates details of the structure and properties of the complex formed between Pt-phenanthroline and the metal binding N-terminal fragment of amyloid-β peptide. Using five distinct starting structures, along with analogous simulations of metal-free peptide, we find that simulations reach equilibration within a few tens of nano-seconds. Equilibrated data collected over more than 800 ns for metal-free and metallated peptides allow detailed comparison of size, secondary structure, and formation of hydrogen bonding and salt bridges.

Small changes in overall size and are observed on Pt binding, but rather larger differences in the mobility of individual residues, measured by root mean square fluctuation, occur. Changes in secondary structure, hydrogen bonding and salt-bridges on complexation of Pt are also observed: in general, His6 and His14 that are bound to Pt are less mobile and more structured than their Pt-free counterparts. Residues between these are slightly more mobile when bound, and exhibit slightly greater propensity to adopt turn and 3–10 helical structures. Hydrogen bonding is reduced by complexation, but salt bridges are more likely to form in the presence of Pt, while close contacts between phenanthrene ligand and aromatic residues Phe4, Tyr10 and His13 are present in at least some of the trajectories, with one structural motif of a sandwich of phenanthrene between Phe4 and His13 observed in a significant proportion of simulation time.

These studies represent first application of ligand field molecular dynamics (LFMD) to address the effect of platinum coordination on amyloid-β structure and flexibility. Previous work from our group concentrated on validating the LFMM method against DFT and the small amount of experimental data available, and on use of conformational searching to examine the effect of variation in ligand structure. Here, we show for the first time that it is possible to quantify the effect of Pt on the dynamical landscape of Aβ configurations adopted, showing that pseudo-equilibration of simulations is possible with relatively modest computational resources. In summary, microsecond timescale molecular dynamics quantifies the subtle but definite changes induced by coordination of Pt(phen) to the N-terminal portion of the amyloid-β peptide. This fragment is widely used as a model for the full, biologically relevant peptides Aβ40 and Aβ42 and their interaction with metal ions: we intend to report analogous data for these peptides in due course.

## Supporting information

S1 Fig Numbering of Pt(phen) region. (TIF)

S2 Fig Starting conformations of free Aβ16 for simulations. Top row: A, B and C. Bottom row: D, E and F.
(TIF)

S3 Fig Superposition of starting conformations of free Aβ16. (TIF)

S4 Fig Overlay of final snapshots of simulations G (grey) and I (yellow). (TIF)

S5 Fig RMSF for Aβ16 simulations. A-E (top) and Pt(Aβ16) simulations F-J (bottom).
(TIF)

S1 Table Merz-Kollman partial charges for Pt(phen)(imid)_2_. (PDF)

S2 Table RMSF details from individual simulations (Å). (PDF)

S3 Table **Percentage of secondary structures for simulations A–J**.
(PDF)
