# Supplementary figures and images for: Ligand field molecular dynamics simulation of Pt(II)-phenanthroline binding to N-terminal fragment of amyloid-β peptide
Source: PLoS One. 2018 Mar 6;13(3):e0193668. doi: 10.1371/journal.pone.0193668 (PMC5839559; doi:10.1371/journal.pone.0193668)

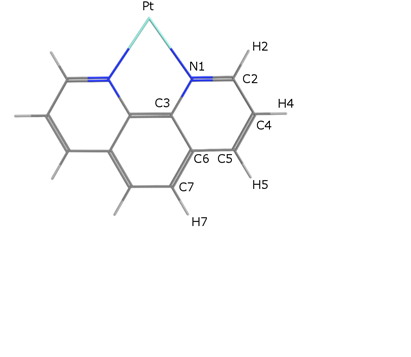

Supplement: S1 Fig — (TIF) [file pone.0193668.s001.tif]

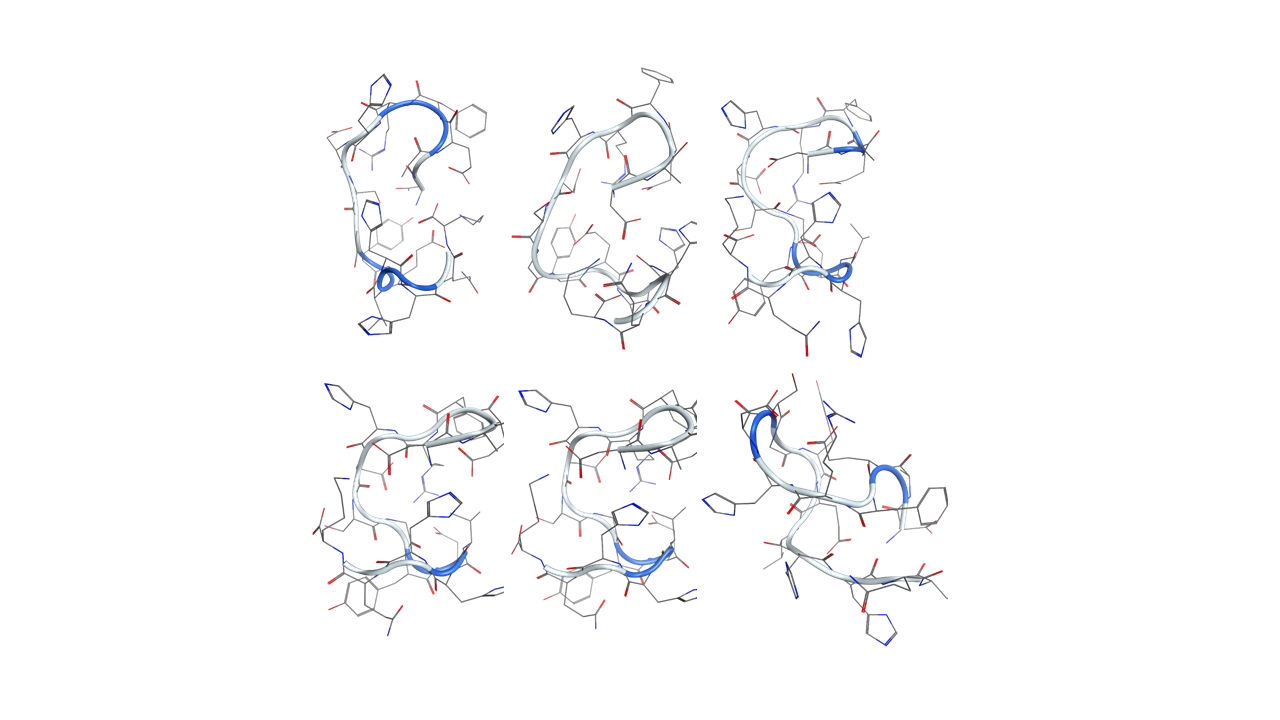

Supplement: S2 Fig — Top row: A, B and C. Bottom row: D, E and F. (TIF) [file pone.0193668.s002.tif]

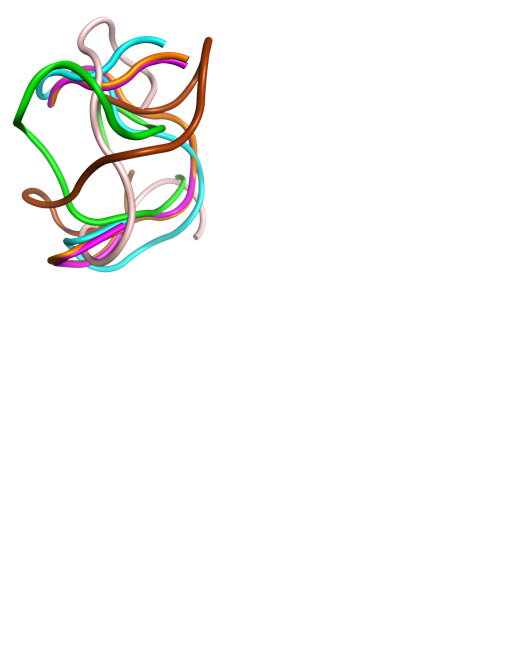

Supplement: S3 Fig — (TIF) [file pone.0193668.s003.tif]

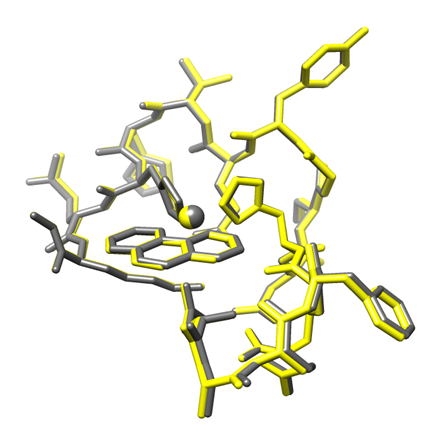

Supplement: S4 Fig — (TIF) [file pone.0193668.s004.tif]

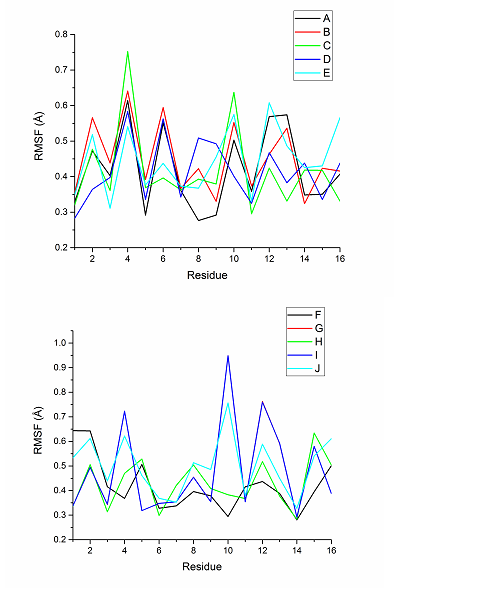

Supplement: S5 Fig — A-E (top) and Pt(Aβ16) simulations F-J (bottom). (TIF) [file pone.0193668.s005.tif]
