# Supplementary material for: Ligand field molecular dynamics simulation of Pt(II)-phenanthroline binding to N-terminal fragment of amyloid-β peptide
Source: PLoS One. 2018 Mar 6;13(3):e0193668. doi: 10.1371/journal.pone.0193668 (PMC5839559; doi:10.1371/journal.pone.0193668)
Supplement: S1 Table — (PDF) [file pone.0193668.s006.pdf]

Table S1: Merz-Kollman partial charges for $Pt(phen)(imid)_2$

**Metal**		**His**		**Phen**	
Atom name	*Charge*	*Atom name*	*Charge*	*Atom name*	*Charge*
Pt	0.560	Nε	0.324	N1	0.025
		Cε	-0.140	C2	0.001
		Nδ	-0.350	C3	0.134
		Cδ	-0.448	C4	-0.262
		Cγ	0.293	C5	-0.017
		Cβ	-0.423	C6	0.077
		Hε	0.248	C7	-0.219
		HδN	0.425	H2	0.190
		HδC	0.267	H4	0.217
		Hβ1	0.160	H5	0.192
		Hβ2	0.162	H7	0.216
