# Supplementary material for: Ligand field molecular dynamics simulation of Pt(II)-phenanthroline binding to N-terminal fragment of amyloid-β peptide
Source: PLoS One. 2018 Mar 6;13(3):e0193668. doi: 10.1371/journal.pone.0193668 (PMC5839559; doi:10.1371/journal.pone.0193668)
Supplement: S2 Table — (PDF) [file pone.0193668.s007.pdf]

Table S2: RMSF Details from individual simulations (Å)

Residue	A	B	C	D	E	Mean	SD
1	0.33	0.35	0.32	0.28	0.35	0.33	0.03
2	0.47	0.57	0.48	0.36	0.52	0.48	0.07
3	0.40	0.44	0.36	0.40	0.31	0.38	0.05
4	0.61	0.64	0.75	0.58	0.54	0.63	0.08
5	0.29	0.39	0.37	0.34	0.38	0.35	0.04
6	0.55	0.59	0.40	0.56	0.44	0.51	0.09
7	0.36	0.37	0.36	0.34	0.37	0.36	0.01
8	0.28	0.42	0.39	0.51	0.37	0.39	0.08
9	0.29	0.33	0.38	0.49	0.46	0.39	0.08
10	0.50	0.55	0.64	0.40	0.57	0.53	0.09
11	0.36	0.37	0.30	0.33	0.33	0.34	0.03
12	0.57	0.46	0.42	0.47	0.61	0.51	0.08
13	0.57	0.54	0.33	0.38	0.49	0.46	0.10
14	0.35	0.32	0.42	0.44	0.43	0.39	0.05
15	0.35	0.42	0.42	0.34	0.43	0.39	0.04
16	0.41	0.42	0.33	0.44	0.57	0.43	0.09
	F	G	H	I	J		
1	0.64	0.34	0.34	0.34	0.53	0.44	0.14
2	0.64	0.49	0.51	0.49	0.61	0.55	0.07
3	0.42	0.34	0.31	0.34	0.44	0.37	0.05
4	0.37	0.72	0.47	0.72	0.62	0.58	0.16
5	0.51	0.32	0.53	0.32	0.46	0.43	0.10
6	0.33	0.35	0.30	0.35	0.37	0.34	0.03
7	0.34	0.35	0.42	0.35	0.35	0.36	0.03
8	0.40	0.45	0.50	0.45	0.51	0.46	0.05
9	0.38	0.36	0.41	0.35	0.49	0.40	0.05
10	0.29	0.95	0.38	0.95	0.76	0.67	0.31
11	0.42	0.35	0.37	0.35	0.38	0.37	0.03
12	0.44	0.76	0.52	0.76	0.59	0.61	0.15
13	0.39	0.59	0.38	0.59	0.45	0.48	0.11
14	0.28	0.29	0.28	0.29	0.33	0.30	0.02
15	0.40	0.58	0.63	0.58	0.54	0.55	0.09
16	0.50	0.39	0.50	0.39	0.61	0.48	0.09
