# Supplementary material for: Ligand field molecular dynamics simulation of Pt(II)-phenanthroline binding to N-terminal fragment of amyloid-β peptide
Source: PLoS One. 2018 Mar 6;13(3):e0193668. doi: 10.1371/journal.pone.0193668 (PMC5839559; doi:10.1371/journal.pone.0193668)
Supplement: S3 Table — Percentage of secondary structures for simulations A–J. (PDF) [file pone.0193668.s008.pdf]

Table S3: Percentage of secondary structures for simulations A – J.

		Turn	β-sheet	Bridge	α-helix	3_{10}-helix	π-helix	Coil
Aβ16	(A)	73.52	0.00	0.07	0.00	4.69	0.00	21.72
	(B)	67.77	0.00	2.57	0.00	0.73	0.03	28.90
	(C)	77.21	0.00	0.00	0.00	0.00	0.00	22.79
	(D)	66.20	0.00	0.00	0.00	0.00	0.00	33.80
	(E)	58.09	0.00	0.00	0.00	0.00	0.00	41.91
Pt(Aβ16)	(F)	41.62	0.00	0.00	0.00	16.35	0.00	42.03
	(G)	82.66	0.00	0.00	0.00	0.00	0.00	17.34
	(H)	55.90	0.00	0.00	0.00	0.00	0.00	44.10
	(I)	82.68	0.00	0.00	0.00	0.00	0.00	17.32
	(J)	53.27	0.00	0.00	0.00	0.00	0.00	46.73
